# Supplementary material for: Symbionts protect aphids from parasitic wasps by attenuating herbivore-induced plant volatiles
Source: Nat Commun. 2017 Nov 30;8:1860. doi: 10.1038/s41467-017-01935-0 (PMC5709398; doi:10.1038/s41467-017-01935-0)
Supplement: Supplementary file 1 — Supplementary Information [file 41467_2017_1935_MOESM1_ESM.pdf]

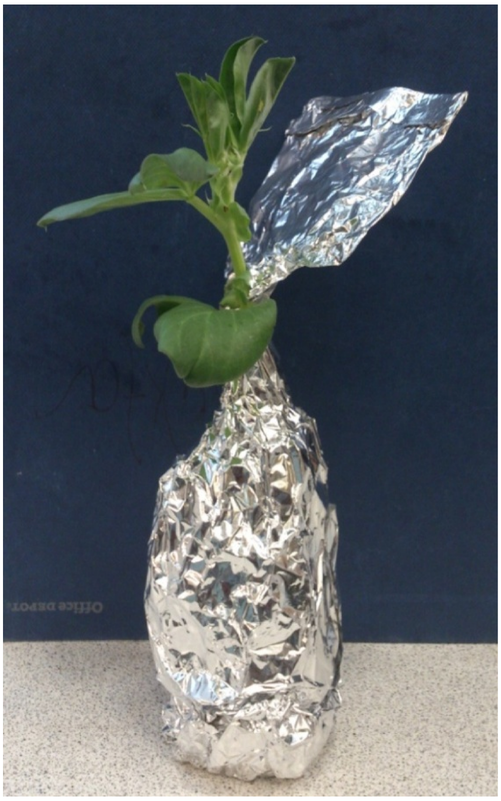

Supplementary Fig. 1
Image of a *Vicia faba* plant with its roots, soil and the leaf onto which clip cages were attached, covered with aluminum foil to test for systemic release of plant volatiles.

Supplementary Table 1

Spearman's rank correlation tests between the relative number of progeny produced by symbiont-carrying and symbiont-free *Acyrthosiphon pisum* aphids during five days on *Vicia faba* plants, and the proportion of time spent by parasitic wasps *Aphidius ervi* above symbiont-infested plants. Each row represents one test with a total of ten plant pairs (n=10). A plot showing these relationships is presented in Fig. 2.

Symbiont species	Symbiont strain	Cured / microinjected	Systemic response	S statistic	p-value	rho
Hamiltonella defensa	101	cured	no	212	0.4274	-0.28
H. defensa	132	cured	no	210	0.4483	-0.27
H. defensa	302	cured	no	170	0.9457	-0.03
H. defensa	132	microinjected	no	204	0.5139	-0.24
H. defensa	404	microinjected	no	156	0.8916	0.05
H. defensa	132	microinjected	yes	162	0.9728	0.02
Regiella insecticola	319	cured	no	142	0.6436	-0.18
R. insecticola	126	cured	no	172	0.9186	-0.04
R. insecticola	319	microinjected	no	186	0.7329	-0.13
R. insecticola	313	microinjected	no	174	0.8916	-0.05
Spiroplasma	227	microinjected	no	154	0.8648	0.07
Spiroplasma	237	microinjected	no	214	0.4070	-0.30
Serratia symbiotica	619	microinjected	no	264	0.0731	-0.60
Rickettsiella	620	microinjected	no	130	0.5599	0.21

Supplementary Table 2

Volatile compounds found in the headspace of *Vicia faba* plants infested with *Acyrthosiphon pisum* aphids carrying the symbiont *Hamiltonella defensa,* or not. Volatiles are ranked based on their variable importance in projection (VIP) in the Principal least squares discriminant analysis (PLS-DA). Volatile identity, type of volatile, its VIP score, retention time and its quantity are shown. Quantities are expressed as peak areas divided by dry plant weight (g). For the compounds with a VIP score larger than 1, pairwise comparisons on volatile quantity between plants carrying the symbiont or not (after log transformation) are also shown (n=9). P-values are corrected with the false discovery rate approach, and significant values are presented in bold. Mean (± SE) quantity of those compounds with a significant difference are represented in Fig. 4. Acetomesitylene: 1,3,5-Trimethyl-2-acetylbenzene, (E)-DMNT: (E)-4,8-Dimethylnona-1,3,7-triene, (E,E)-TMTT: (E,E)-4,8,12-Trimethyltrideca-1,3,7,11-tetraene, ar: Aromatic.

Supplementary Table 2 (cont.)

Compound	Id	Class	Ret. time (min)	VIP score	log (quantity) in Symbiont present	Symbiont removed	p-value
Germacrene D	49	Terpenoid	21.43	1.6691	14±0.18	15.2±0.19	**0.0084**
Acetomesitylene	29	ar-Ketone	17.53	1.5813	12.61±0.16	13.3±0.11	**0.0234**
Nerolidol	52	Terpenoid	21.94	1.5484	7.02±1.78	10.95±0.26	0.0758
β-Cubebene	35	Terpenoid	19.47	1.5140	12.39±0.08	13.09±0.16	**0.0148**
3,4-Xylenol	27	Phenol	14.51	1.4906	15.2±0.26	16.17±0.19	**0.0304**
2,4-Dimethyl furan	2	Ether	3.72	1.3694	15.54±0.18	16.27±0.19	**0.0371**
Aromadendrene	45	Terpenoid	21.02	1.3560	10.04±0.27	11.09±0.19	**0.0304**
Unknown	21	NA	12.95	1.3455	14.88±0.1	15.51±0.2	**0.0371**
β-Copaene	40	Terpenoid	20.33	1.2981	12.77±0.22	13.66±0.2	**0.0304**
α-Amorphene	51	Terpenoid	21.80	1.2720	12.32±0.21	13.13±0.19	**0.0371**
2-Acetyl-3,5-dimethylfuran	19	Ketone	12.42	1.2445	14.91±0.23	15.59±0.16	0.0586
2,4-Pentanedione	3	Ketone	4.84	1.1952	15.77±0.5	16.84±0.25	0.1002
β-Myrcene	10	Terpenoid	9.82	1.1512	14.7±0.16	14.6±0.1	0.6406
Isogermacrene D	46	Terpenoid	21.07	1.1321	12.97±0.22	13.65±0.19	0.0755
(*E,E*)-2,4-Hexadienal	6	Aldehyde	6.98	1.1214	12.27±0.33	13.18±0.25	0.0758
3-Heptanone	7	Ketone	7.17	1.1089	12.07±0.14	12.54±0.22	0.1221
Unknown	33	NA	19.23	1.1087	11.19±0.35	12.3±0.45	0.1002
α-Cadinene	56	Terpenoid	22.57	1.0895	10.84±0.31	11.67±0.22	0.0758
Unknown sesquiterpene	50	Terpenoid	21.67	1.0756	12.36±0.22	13±0.21	0.0824
γ-Cardinene	53	Terpenoid	22.09	1.0278	12.48±0.26	13.14±0.23	0.1002
Methyl dihydrojasmonate	61	Ester	24.74	1.0140	12.69±0.4	13.29±0.29	0.2786
β-Atlantol	59	Terpenoid	23.79	1.0123	12.39±0.31	12.9±0.35	0.3177
Diethyl adipate	34	Ester	19.28	1.0009	9.7±0.54	10.88±0.75	0.2647
α-Caryophyllene	44	Terpenoid	20.86	1.0002	15.26±0.15	15.33±0.14	0.7372
δ-Cadinene	54	Terpenoid	22.26	0.9984	12.94±0.22	13.44±0.19	NA
(*E,E*)-TMTT	58	Terpenoid	23.34	0.9981	17.46±0.15	17.84±0.19	NA
Unknown	64	NA	30.01	0.9947	13.07±0.3	13.06±0.25	NA
β-Caryophyllene	39	Terpenoid	20.14	0.9817	16.12±0.16	16.05±0.14	NA
(*E*)-α-Bergamotene	41	Terpenoid	20.41	0.9810	14.02±0.15	13.97±0.24	NA
2,2,6-Trimethylcyclohexanone	15	Ketone	10.93	0.9796	13.18±0.12	13.64±0.21	NA
Chiloscyphone	57	Terpenoid	22.67	0.9793	13.54±0.21	14.12±0.29	NA
(*Z*)-α-Bergamotene	38	Terpenoid	20.01	0.9776	12.54±0.16	12.55±0.19	NA
3,3-Dimethyl-2,4-pentanedion	8	Ketone	7.82	0.9695	12.14±1.53	14.08±0.39	NA

Supplementary Table 2 (cont.)

Compound	Id	Class	Ret. time (min)	VIP score	log (quantity) in Symbiont present	log (quantity) in Symbiont removed	p-value
Geranyllinalool	65	Terpenoid	30.46	0.9667	13.12±0.26	13.09±0.38	NA
α-Copaene	32	Terpenoid	19.17	0.9449	12.76±0.1	12.97±0.11	NA
β-Springen	63	Terpenoid	29.36	0.9408	13.51±0.14	13.96±0.23	NA
(*E*)-Cadina-1,4-diene	55	Terpenoid	22.46	0.9266	11.36±0.16	11.64±0.17	NA
γ-Muurolene	48	Terpenoid	21.32	0.9094	12.1±0.22	12.54±0.23	NA
Clovene	31	Terpenoid	18.82	0.9052	11.14±0.27	11.66±0.21	NA
4-Isopropylcyclohexanol	23	Alcohol	13.24	0.8891	11.96±1.51	10.29±1.97	NA
Linalool	20	Terpenoid	12.61	0.8778	13.55±0.3	13.72±0.31	NA
(*Z*)-3-Hexen-1-ol, acetate	12	Ester	10.25	0.8555	17.95±0.18	18.17±0.11	NA
β-Funebrene	36	Terpenoid	19.61	0.8416	9.14±1.19	10.92±0.32	NA
(*Z*)-β-Ocimene	16	Terpenoid	11.03	0.8370	15.44±0.16	15.44±0.13	NA
(*E*)-Cadina-1(6),4-diene	47	Terpenoid	21.25	0.8276	9.19±0.27	7.52±1.44	NA
Alloocimene	24	Terpenoid	13.32	0.8256	13.29±0.19	13.3±0.16	NA
(*E*)-β-Ocimene	17	Terpenoid	11.35	0.8062	18.22±0.12	18.28±0.1	NA
Geranylgeraniol	66	Terpenoid	30.50	0.8046	14.03±0.13	14.38±0.23	NA
(*Z*)-3-Hexen-1-ol	5	Alcohol	6.47	0.8044	16.65±0.19	16.79±0.13	NA
Limonene	14	Terpenoid	10.79	0.7775	14.7±0.15	14.7±0.13	NA
(*E*)-DMNT	22	Terpenoid	13.00	0.7410	16.14±0.21	16.25±0.24	NA
(*E*)-β-Ocimene epoxide	26	Terpenoid	13.65	0.7395	14.25±0.21	14.56±0.2	NA
Unknown	60	NA	24.33	0.7359	10.6±1.35	12.09±0.26	NA
(*E,E*)-Cosmene	25	Terpenoid	13.34	0.6993	14.5±0.19	14.61±0.11	NA
Anisole	9	ar-Ether	7.96	0.6942	13.69±0.28	14.15±0.17	NA
1,5,8-p-Menthatriene	18	Terpenoid	12.09	0.6938	12.71±0.2	12.88±0.11	NA
Unknown	11	NA	10.20	0.6857	12.87±0.18	12.91±0.1	NA
(*Z*)-Lanceol acetate	62	Ester	28.35	0.6684	14.15±0.11	14.47±0.27	NA
1-Butanol	1	Alcohol	3.10	0.6552	15.68±0.22	16.06±0.25	NA
(*Z*)-Jasmone	37	Ketone	19.63	0.6488	10.05±1.29	11.43±0.25	NA
α-Cubebene	30	Terpenoid	18.57	0.6145	9.53±1.22	10.52±0.12	NA
Methyl salicylate	28	ar-Ester	15.01	0.6035	15.65±0.25	15.96±0.29	NA
(*E*)-β-Farnesene	43	Terpenoid	20.82	0.6004	14.18±0.13	14.42±0.27	NA
(*E*)-2-Hexenal	4	Aldehyde	6.39	0.5831	12.7±0.26	12.94±0.25	NA
Sesquisabinene	42	Terpenoid	20.56	0.5243	11.87±0.17	12.01±0.27	NA
3-Methylanisole	13	ar-Ether	10.57	0.2948	13.15±0.22	13.25±0.17	NA
